# The Molecular Signature of Human Testicular Peritubular Cells Revealed by Single-Cell Analysis

**DOI:** 10.3390/cells11223685

**Published:** 2022-11-19

**Authors:** Annika Liebich, Nina Schmid, Christina Koupourtidou, Carola Herrmann, Kim-Gwendolyn Dietrich, Harald Welter, Jovica Ninkovic, Artur Mayerhofer

**Affiliations:** 1 Biomedical Center, Cell Biology, Anatomy III, Faculty of Medicine, Ludwig Maximilian University of Munich, 82152 Planegg-Martinsried, Germany; 2 Helmholtz Center Munich, Institute of Stem Cell Research, 85764 Neuherberg, Germany

**Keywords:** human testis, cellular model, fertility, cellular plasticity

## Abstract

Peritubular cells of the human testis form a small compartment surrounding the seminiferous tubules. They are crucial for sperm transport, and they emerge as contributors to the spermatogonial stem cell niche. They are among the least known cell types of the human body. We employed single-cell RNA sequencing of cultured human testicular peritubular cells (HTPCs), which had been isolated from testicular samples of donors with normal spermatogenesis. The significant overlap between our results and recently published ex vivo data indicates that HTPCs are a highly adequate cellular model to define and study these cells. Thus, based on the expression of several markers, HTPCs can be classified as testicular smooth muscle cells. Small differences between the in vivo/in vitro expressed genes may be due to cellular plasticity. Plasticity was also shown upon addition of FCS to the culture medium. Based on transcriptome similarities, four cellular states were identified. Further analyses confirmed the presence of known stem cell niche-relevant factors (e.g., GDNF) and identified unknown functions, e.g., the ability to produce retinoic acid. Therefore, HTPCs allow us to define the signature(s) and delineate the functions of human testicular peritubular cells. The data may also serve as a resource for future studies to better understand male (in)fertility.

## 1. Introduction

Understanding the cellular components of the human testis is of importance for the understanding of the testis, male fertility and infertility. Furthermore, with regard to male hypogonadism, such insights are required to guide regenerative medicine [1,2].

Recent studies have begun to unravel the cellular components of the human testis at the single-cell level [3,4,5,6]. These single-cell RNA sequencing (scRNAseq) studies have provided important insights into the molecular nature of the most abundant cells, specifically germ cells and somatic testicular cells, i.e., Leydig and Sertoli cells. 

They also provide some details about the least known cell type of the human testis, peritubular cells [7,8], which remain, however, to be fully defined with respect to its cellular phenotype(s), plasticity and functions. Several layers of these cells together with the extracellular matrix form the peritubular compartment in males, while only one layer of peritubular cells is found in rodent species. Originally based on electron microscopical characteristics, they were described as smooth muscle cells (SMCs) [9], yet their specific cellular signature is not fully known. 

Of note, in states of male infertility, profound changes of human peritubular cells have been noted [3], and these results are in line with earlier studies [10,11,12,13,14,15]. This indicates phenotypic switching, a process that is not fully understood, and also occurs in other SMCs, especially vascular SMCs in the context of atherosclerosis [16]. 

As these cells form a small compartment within the testis, the mentioned previous ex vivo analyses are based on a few hundred peritubular cells (e.g., 900 cells in Nie et al. [17] from four patients; 550 cells in Di Persio et al. [3] from one patient), a limitation that, as we have reasoned, can be overcome by studying cultured cells, provided that they reflect the in situ situation and retain their phenotype. 

Previous studies have indicated that cultured human testicular peritubular cells (HTPCs) [18] closely resemble their respective in situ-counterparts [18,19,20], which is an assumption that is based on a number of markers expressed both in vivo and in situ (e.g., ACTA2, DCN, BGN, AR and NR3C1). Indeed, SMCs derived from different anatomical locations, while having distinct expression patterns, have been reported to retain their expression profiles even after serial passaging in vitro [21]. 

We studied cultured HTPCs and performed single-cell RNA seq analyses. We compared the results with own data, including proteomic results, the in situ protein data available in the Human Protein Atlas (https://www.proteinatlas.org; accessed on 1 April 2022), and transcriptomic information available (Nie et al. [17], selection of young patients; Di Persio et al. [3]). Based on results, which indicated the expression of mesenchymal stromal cell (MSC) characteristics and hints of retinoic acid synthesis, we performed additional studies to explore the plasticity and differentiation potential of HTPCs and we examined their ability to produce retinoic acid.

## 2. Materials and Methods

### 2.1. HTPCs Culture

**Figure 1:**
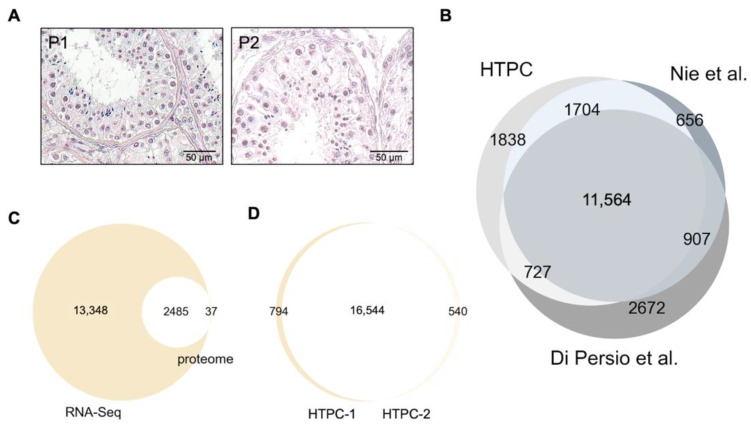
(**A**): H.&E. staining of seminiferous tubules of testes samples of which HTPC-1 (P1) and HTPC-2 (P2) were derived from. They show ongoing spermatogenesis and normal morphology of the tubular wall. (**B**): Venn diagram of in vitro data of HTPCs (HTPC-1, no FCS) and previously published ex vivo data [3,17]. Please, note that the extent of the differences between the two ex vivo datasets are similar to the differences between our data and the ex vivo data. (**C**): Overlap of scRNAseq data with proteomic data of the same donor [22]. (**D**): Similarity of HTPC-1-derived and HTPC-2-derived transcripts (14,916 common transcripts and 324 or 917, respectively, unique ones).

We examined the HTPCs, which were derived from the small testicular samples of two donors (P1 and P2; HTPC-1 from P1: 41 years old, and HTPC-2 from P2: 48 years old) with obstructive azoospermia and normal spermatogenesis [22]. Small parts of each were also fixed and embedded in paraffin. They allowed us to examine spermatogenesis and architecture of the peritubular wall. Both of them were normal (Figure 1). The methods have been described previously [18,22,23]. The cells from passages 8 (HTPC-1) and 5 (HTPC-2) were studied. They were maintained and propagated in Dulbecco’s modified Eagle Medium (DMEM) containing high glucose (4.5 g/L; Gibco, Paisley, UK) with 10% (*v*/*v*) fetal calf serum (FCS) (Capricorn Scientific, Ebsdorfergrund, Germany) and 1% (*w*/*v*) penicillin/streptomycin (P/S; Gibco, Paisley, UK) at 37 °C, 5% CO_2_ and 95% humidity.

### 2.2. Single-Cell RNA Sequencing (scRNAseq)

For the single-cell RNA sequencing analysis, the HTPCs were cultured for 24 h with or without the supplementation of 10% FCS in the medium. The addition of FCS fosters proliferation and allows for the expansion and propagation of the HTPCs. The cells were trypsinized, washed with PBS, centrifuged, resuspended in PBS, filtered through a cell strainer (mesh size = 40 µm) and counted (CASY^®^ Cell Counter, OMNI Life Science, Bremen, Germany). The cells were prepared to a concentration of ~1000 cells/µL in PBS 0.04% BSA for the single-cell sequencing and processed using the Single-Cell 3′ Reagent Kits v2 from 10x Genomics according to the manufacturer’s instructions, targeting 10,000 cells. This was followed by the GEM generation and barcoding, post-GEM-RT cleanup, cDNA amplification, library construction and quality assessment using the Bioanalyzer (Agilent, Santa Clara, CA, USA). Illumina sequencing libraries were sequenced using a NovaSeq 6000 (NovaSeq Flow cell Type S2 one lane), with an average read depth of 50,000 aligned reads per cell. The sequencing was performed in the genome analysis center of the Helmholtz Center Munich.

### 2.3. Alignment and Data Analysis

The transcriptome alignment of single-cell data was performed using CellRanger 4.0.0 against the human transcriptome GRCh38. The Quality Control (QC) of the mapped cells was performed using the recommendations by Luecken and Theis [24]; we selected ~6500 cells with at least 6000 UMI counts, 1600 detected genes and mitochondrial gene counts lower than 10%. The doublets were removed using the Scrublet framework [25]. The normalization and log transformation were performed using the scran package normalize_total and log1p functions, respectively [26]. A highly variable gene selection was performed via the function highly_variable_genes using the Cell Ranger flavor with a fault parametrization, and we obtained 5000 highly variable genes in at least one experimental group [27]. Following the cell count normalization and scaling (function scale in SCANPY), the experimental groups were integrated with Scanorama [28]. The unsupervised clustering of cells was performed using the Leiden algorithm [29], as implemented in SCANPY, and with resolution parameters of 0.01 (HTPC-1) and 0.02 (HTPC-2). This allowed for the classification and counting of the main clusters based on the marker genes that were selected using test_overestim_var between the normalized counts of each marker gene in a cluster against all of the others (function rank_genes_groups in SCANPY). The visualization of the cell groups was performed using Uniform Manifold Approximation and Projection (UMAP) [30], as implemented in SCANPY. The cell cycle genes were scored according to Tirosh et al. [31], and the appropriate phase that defined each single cell was identified using the function score_genes_cell_cycle in SCANPY.

### 2.4. RNA Velocity

To generate the spliced/un-spliced expression matrices, we used the tool velocyto using the function run10x in order to directly accomplish the counting in the cellranger output folder [32]. To define the cellular dynamics, differentiation and related trajectories, we used the scVelo toolkit [33]. The calculated velocity was projected and embedded onto the PAGA graph to show the connectivity of the adjacent clusters.

### 2.5. Comparison with Existing Ex Vivo and In Situ Data

To control for the occurrence of cell culture-related phenomena, we turned to the images that are provided in the Human Protein Atlas (https://www.proteinatlas.org, accessed on 1 April 2022) to examine in situ expression. Because the human samples that had been used for the immunostaining and are depicted there do not necessarily represent healthy tissues, we used the images only when the signs of normal spermatogenesis were visible (e.g., presence of sperm heads; see Appendix B; Table A1 for a list of the images that we used).

In addition, we mined the publicly available human testis scRNAseq data (Nie et al. [17]; NIH Gene expression Omnibus (GEO): GSE182786), which contain information about young adult men (17–22 years old). We also examined the publicly available data (Di Persio et al. [3]; NIH Gene expression Omnibus (GEO): GSE153947), which contain information on testicular cells from men with obstructive azoospermia. Please note that for the comparison, we used the data from a man (N3; 55 years old) which closely matched the donors of our cells with respect to age and obstructive azoospermia. Of note, similar to our study, Nie et al. [17] and Di Persio et al. [3] used the same platform (10x Genomics). 

### 2.6. Comparison with Proteomic Data

The cells from HTPC-1, which were cultured without FCS for 24 h, were previously examined by mass spectrometry in one of our studies [22]. The results, which are previously presented in the frame of a larger study, were re-evaluated and compared.

### 2.7. Differentiation Studies

We studied additional HTPCs (*n* = 2; patient were 29 years old) to explore the ability of these cells to differentiate into osteoblasts and adipocytes. We used reagents from Miltenyi Biotec GmbH (Bergisch Gladbach, Germany), following the instructions of the manufacturer (StemMACSTM AdipoDiff Media/OsteoDiff Media; #130-091-677/#130-091-678). After a period of 14 or 21 days, respectively, alkaline phosphatase/oil red (SIGMA FASTTM BCIP^®^/NBT Buffered Substrate Tablet (Sigma #B5655); Oil Red O (Sigma #O9755)) was used to visualize the success of the differentiation. Furthermore, a qPCR for the typical osteoblast (*ALPL/COMP*) or adipocyte (*PLIN1*) markers was performed. The information about the primers that were used is given in the Appendix B; Table A2. As we found an abundant expression of *TCF21* in the FCS-treated HTPCs, we also addressed the question whether Leydig cells could be derived from HTPCs, following a recently published protocol [34]. We studied additional HTPCs (*n* = 2; patients were 29 and 31 years old). In brief, HTPCs were seeded on cell culture dishes and initially recovered for 18 h in DMEM + 10 % FCS. Subsequently, the cells were expanded for 3 days in DMEM/F12 which was supplemented with Normocin (Invivogen, Toulouse, France), 10 ng/mL PDGFAA Abcam, Cambridge, UK), 10 ng/mL PDGFBB (Abcam), 0.5 μM SAG (Biomol, Hamburg, Germany) and 10 ng/mL FGF2 (Biomol). After 3 days, the expansion media were replaced with differentiation media: DMEM/F12 which was supplemented with penicillin/streptomycin, 10 ng/mL PDGFAA, 10 ng/mL PDGFBB, 0.5 μM SAG, 10 ng/mL FGF2, 5 mM LiCl2 (Merck, Darmstadt, Germany) and 10 μM DAPT (Biomol). After 10 days in differentiation media, the cells were analyzed for their Leydig cell properties by qPCR. The expressions of *INSL3*, *LHR* and steroidogenic enzymes were used as readout (Appendix B; Table A4).

### 2.8. ELISA Test for Retinoic Acid

Because we found components of the retinoic acid synthetic pathway in the transcriptomic data, we examined whether the HTPCs produce retinoic acid, employing additional HTPCs (*n* = 2; 36 and 38 years old) which were cultured with and without FCS. The detection of retinoic acid was performed using an ELISA (Human retinoic acid ELISA Kit; MBS167278, MyBiosource, San Diego, CA, USA) following the instructions of the manufacturer.

## 3. Results

### 3.1. General Remarks

**Figure A1:**
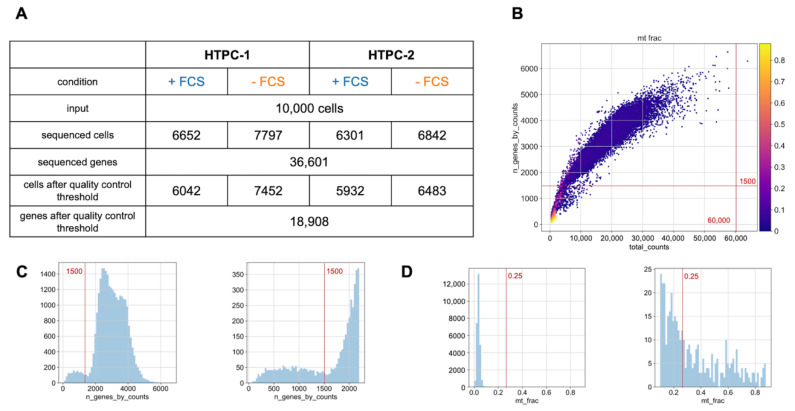
(**A**): Summary of cell numbers of both HTPCs and the amounts of detected transcripts. (**B**–**D**): Information about quality control of single-cell RNA-seq data. Thresholds are displayed in red lines. (**B**) Number of detected genes according to count depth per cell. The color code indicates the fraction of mitochondrial counts. Quality thresholds were set to a minimum of 1500 genes and a maximum cell depth of 60,000. (**C**) Number of genes per cell. The right graph shows counts in less than 2500 genes. (**D**) The minimum mitochondrial fraction was set to 0.25.

The results of the single-cell RNA sequencing analyses of the HTPCs from two donors yielded close to 18,000 identified (Appendix A; Figure A1A). In Figure 1A, the morphology of the seminiferous tubules of testes samples of HTPC-1/HTPC-2 with ongoing spermatogenesis and no obvious alteration of the tubular wall is depicted.

The similarity between the HTPC-1-derived and HTPC-2-derived cells is high (16,544 common transcripts and 794 or 540, respectively, unique ones). We focused on the sample derived from the HTPC-1 group (younger man; 41 years old) because it appeared to be most comparable to the samples of the recent ex vivo studies [3,17]. Further, this allowed for the comparison of the scRNAseq data with the proteomic data of the same donor [22]. This comparison between the proteome data and transcriptome data revealed that of 37 proteins, which had been previously identified, the transcripts were not represented in the transcriptome data, while 2485 of them were readily found (Figure 1C,D). 

We compared the data of HTPC-1 (no FCS) with the ex vivo data [3,17]. To compare our in vitro-derived sample to the ex vivo studies, we focused on the serum-starved cells (i.e., without FCS) because these non-proliferative cells are more comparable to the peritubular cells within the testis. Figure 1B shows that a large overlap that exists between the three data sets (58%). The differences between the two ex vivo data sets are similar to the differences between our data and the ex vivo data.

**Figure 2:**
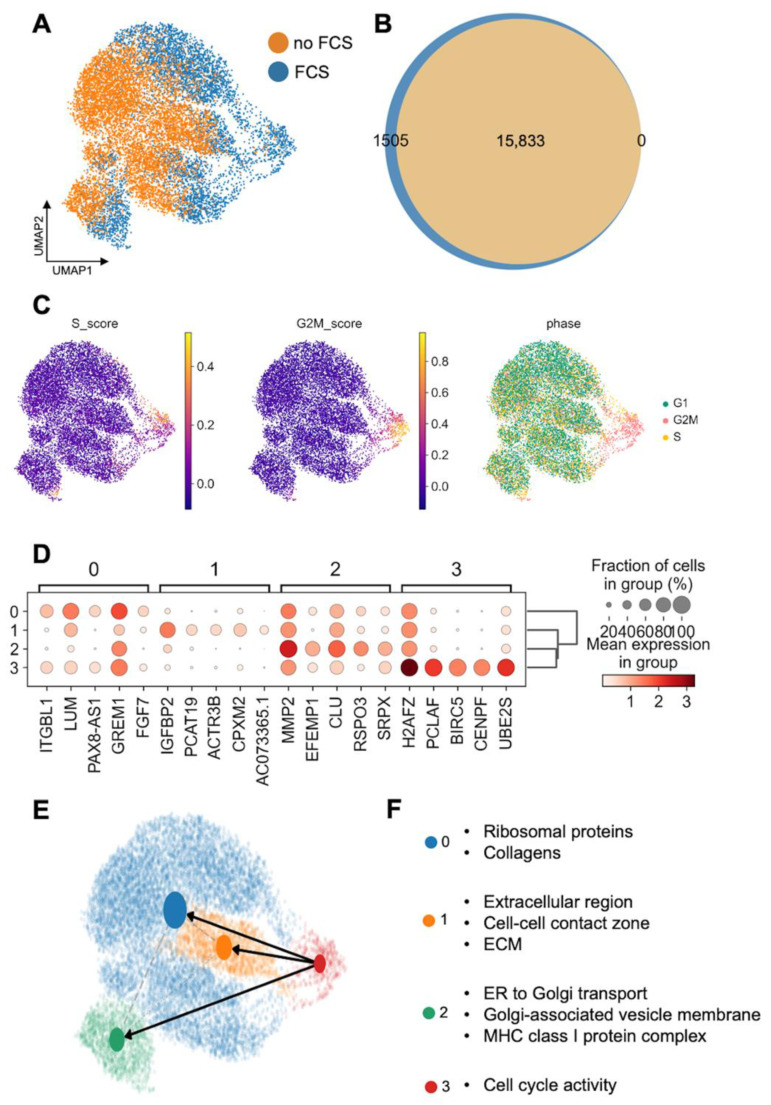
(**A**): UMAP plot of samples derived from HTPC-1 with and without FCS. (**B**): Venn diagram indicates overlap of both of the samples. The exposure of HTPCs to FCS results in the expression of 1505 additional genes. (**C**): Cell cycle stages [31] display one distinct proliferating cluster in the FCS sample. (**D**–**F**): Four distinct states of cellular differentiation were observed, indicating plasticity and proliferation. (**D**) Dot plot demonstrates expression of most abundant genes in each cluster. (**E**) Velocity analysis was embedded into the PAGA graph, which indicates trajectory of the proliferating cluster into three functional subgroups. (**F**) Representative GO terms after string analysis with upregulated genes.

**Figure A3:**
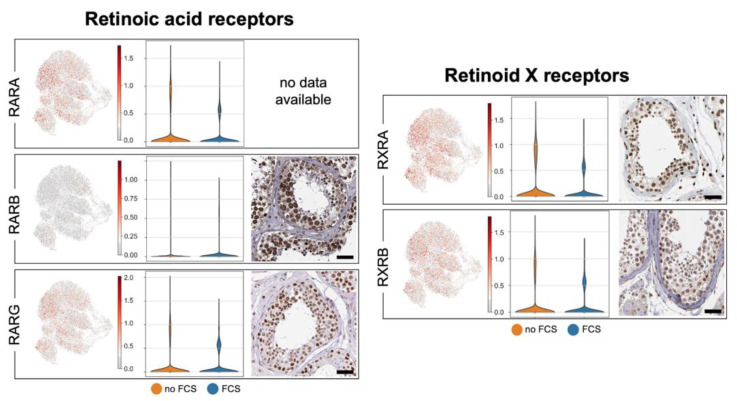
Summary transcript information of retinoic acid and retinoid X receptors. UMAP plots in HTPCs (**left side**). Violin plots, comparing expression with and without FCS of HTPC-1, (**middle**) and corresponding protein detection with HPA-images (**right side**; scale bars = 50 µm). Note that abundance of some transcripts are affected by FCS.

The addition of FCS induced cell proliferation, as expected. Four distinct states of cellular differentiation were identified upon the analysis, indicating the plasticity of these cells (Figure 2). Some differences between HTPC-1 and HTPC-2 became apparent upon velocity analyses (Figure 2 and Appendix A, Figure A3).

The data obtained provide a thorough molecular signature of the human testicular peritubular cells. Here, we focus on the key results. We show in the following sections, the results that were obtained from the study of cells of HTPC-1, unless they are indicated otherwise.

### 3.2. Human Testicular Peritubular Cells Are a Testicular Subtype of Smooth Muscle Cells

**Figure 3:**
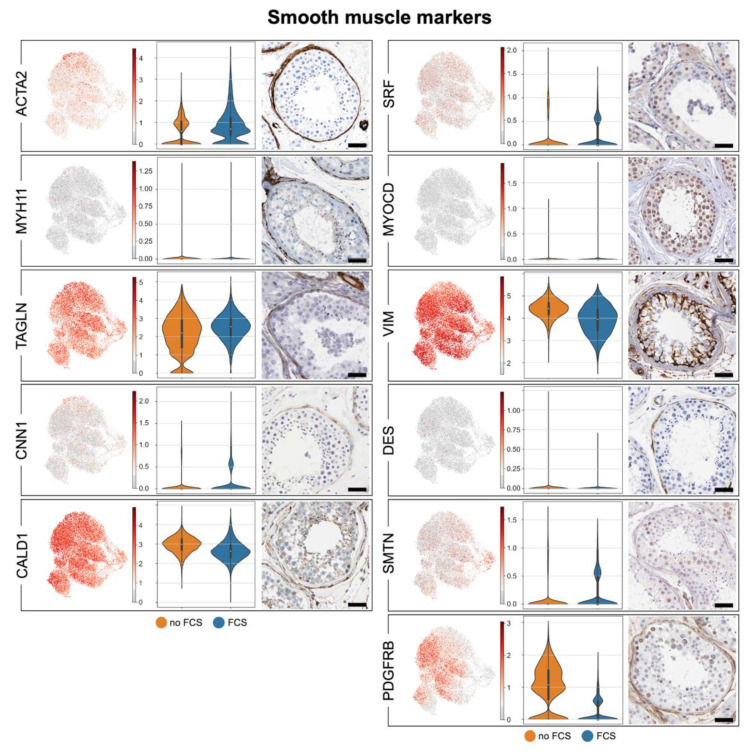
Smooth muscle markers. UMAP plots of prototype smooth muscle markers in HTPCs (**left side**). Violin plots comparing expression with and without FCS of HTPC-1 (**middle**) and corresponding protein detection with HPA images (**right side;** scale bars = 50 µm). Please, note that abundances of some transcripts are affected by FCS. A comparison between HTPCs and ex vivo data is provided in Table 1. The markers shown here are also seen in testicular vascular SMCs; see Table A1 for links to the respective HPA images.

The contractile abilities, i.e. a key feature of SMCs, are retained by the isolated HTPCs [19,35], and HTPCs were described to structurally resemble SMCs [9]. A number of distinct SMCs exist, which share typical marker combinations, as it has been recently reviewed [36]. The robust presence of the prototype SMC markers *ACTA2*, *VIM*, *SM22A* (*TAGLN*), calponin (*CNN1*), caldesmon (*CALD1*) and smoothelin (*SMTN*) were not or only marginally affected by the absence/presence of FCS, and this reveals the SMC character of the HTPCs (Figure 3).

Additional SMC markers [36], namely, *SMMHC* (*MYH11*), desmin (*DES*), myocardin (*MYOCD*), and serum response factor (*SRF*) or (not shown) *CSPG4*, *RGS5*, *EZH2* and *LGR5,* were expressed only by a few subtypes of HTPCs, and they did not notably change upon FCS addition to the medium. *PDGFRB*, which is found in vascular SMCs, but not in airway or intestinal SMCs [36], was present in the HTPCs. Of note, this receptor is also considered to be a marker for myofibroblasts [37], and it was increased in the absence of FCS. The corresponding proteins were represented in the peritubular cells in situ as indicated by images provided by the Human Protein Atlas. Table 1 depicts a comparison of our data (HTPC-1/HTPC-2; no FCS) with the ex vivo data of Di Persio et al. [3] and Nie et al. [17].

### 3.3. Human Testicular Peritubular Cells Are Producers of ECM, Which Is a Constituent of the Peritubular Wall

**Figure 4:**
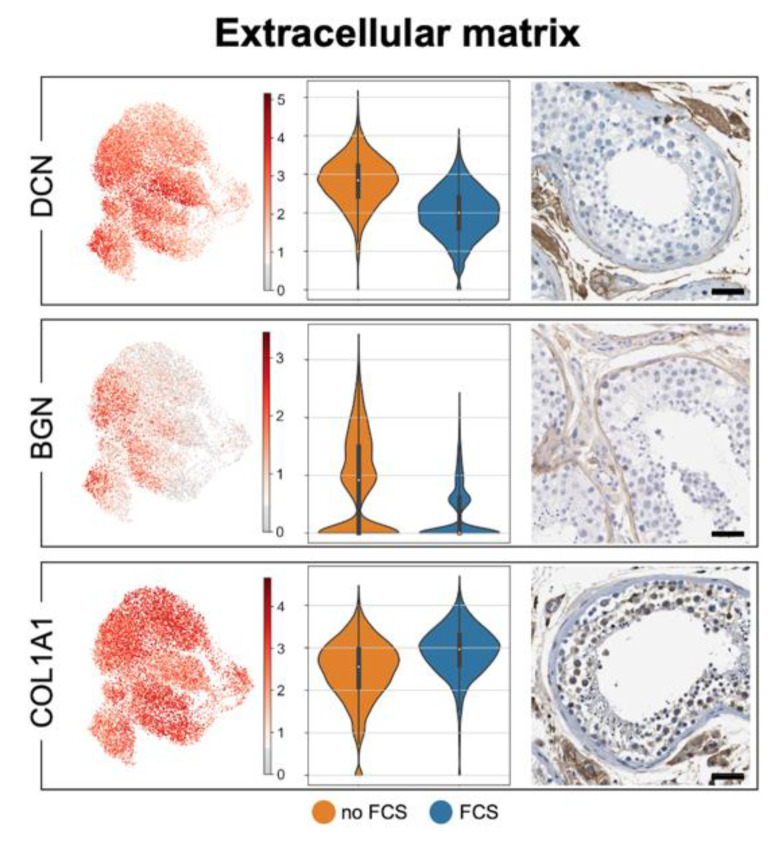
ECM components. UMAP plots of ECM components produced by HTPCs and typically found in the peritubular wall, namely, *BGN*, *DCN* and *COL1A1* (**left side**). Violin plots comparing expression with and without FCS of HTPC-1 (**middle**) and corresponding protein detection with HPA images (**right side**; scale bars = 50 µm). Please, note that *BGN* and *DCN* levels are increased in the absence of FCS in the culture medium. A comparison between HTPCs and ex vivo data is provided in Table 1.

Known ECM components of the human peritubular wall compartment include DCN, BGN and COL1A1, among others [10,35,38]. Figure 4 shows that *BGN* and *DCN* expression levels are increased in the absence of FCS in the culture medium. The corresponding proteins are represented in the peritubular cells in situ (Human Protein Atlas). These ECM components were also previously readily found, specifically, in the secretome of HTPCs [39]. Hence, they are produced by HTPCs, and their production is regulated. Table A3 (Appendix B) shows the comparison of our data (HTPC-1/HTPC-2; no FCS) with the data of Di Persio et al. [3] and Nie et al. [17].

### 3.4. Human Testicular Peritubular Cells Are Targeted by Hormones and Local Factors: Receptors and Ion Channels

**Figure 5:**
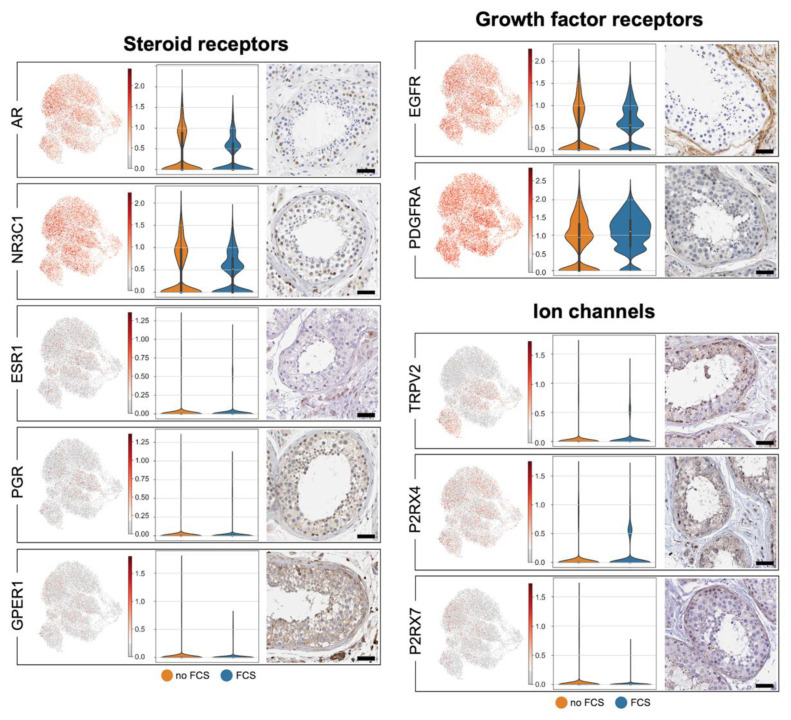
Receptors and ion channels. UMAP plots of previously described receptors and ion channels of HTPCs (**left side**). Violin plots comparing expression with and without FCS of HTPC-1 (**middle**) and corresponding protein detection with HPA images (**right side**; scale bars = 50 µm). No distinct differences in the expression levels are apparent when FCS was absent in the culture medium. A comparison between HTPCs and ex vivo data is provided in Table A3 (Appendix B).

The known receptors involved in the regulation of HTPCs are AR and NR3C1, which mediate the actions of androgens and glucocorticoids, respectively [35,40]. Figure 5 shows that *AR* and *NR3C1* were homogeneously expressed, as were *EGFR* [10] and *PDGFRA* [41]. Further, the steroid receptors *PGR* and *ESR1* were found, as well as the ion channels *TRPV2* [42] and *P2X4/7* [20]. The corresponding proteins in the peritubular cells in situ are indicated by the images that were provided by the Human Protein Atlas. Table A3 (Appendix B) shows the comparison of our data (HTPC-1/HTPC-2; no FCS) with the data of Di Persio et al. [3] and Nie et al. [17].

### 3.5. Human Testicular Peritubular Cells Are a Source of Factors Involved in the Regulation of Spermatogenesis

**Figure 6:**
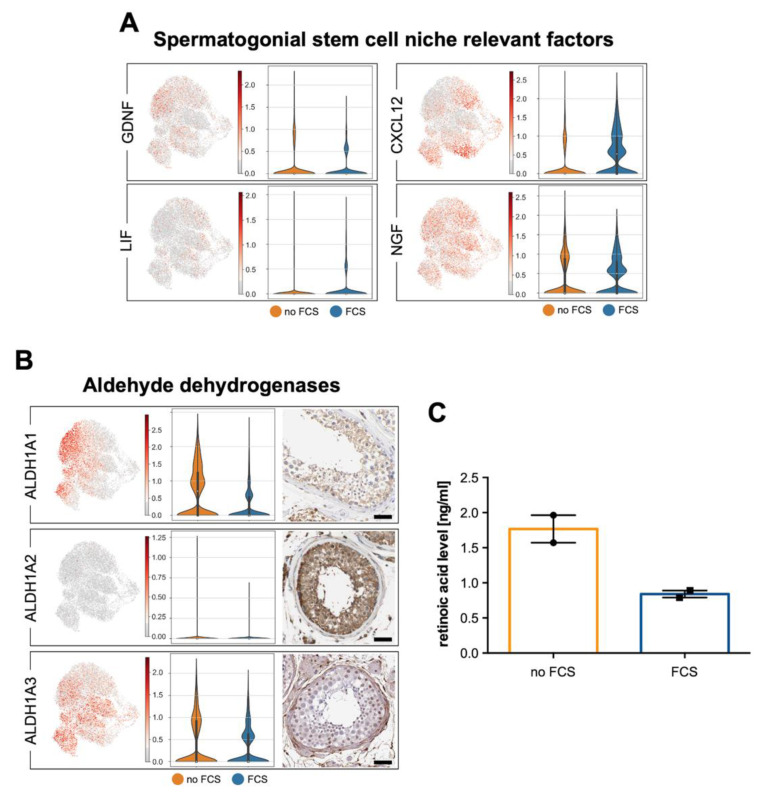
Summary of factors relevant for spermatogenesis. (**A**) UMAP plots of *GDNF*, *LIF*, *CXCL12* and *NGF* in HTPCs (**left side**) and corresponding violin plots comparing expression with and without FCS of HTPC-1 (**right side**). (**B**) Transcript detection of the aldehyde hydrogenases *ALDH1A1* and *ALDH1A3*. Violin plots comparing expression with and without FCS of HTPC-1 (**middle**) and corresponding protein detection with HPA images (**right side**; scale bars = 50 µm). ALDH1A2 is not expressed in peritubular cells, in vitro or in situ. A comparison between HTPCs and ex vivo data is provided in Table A3 (Appendix B) (**C**) Result of retinoic acid level in HTPCs culture medium (2 samples). Please note a decrease in the presence of FCS in the culture medium.

The factors that are produced by murine testicular peritubular cells and HTPCs can influence the SSC (e.g., GDNF) and spermatogenesis [43,44]. In the HTPCs, heterogeneity and weak regulatory influences of FCS were found for *GDNF*, *CXCL12*, *LIF* and *NGF*, as well as for *ALDH1A1* and *ALDH1A3* (Figure 6). GDNF, CXCL12 and NGF production were previously verified [40,44], and they showed variability with regard to their production by different patient-derived HTPCs. At this point in the study, we addressed the possibility that retinoic acid may be derived from HTPCs. The biosynthetic enzymes (ALDH1A1 and ALDH1A3) are also represented by the HTPCs in situ (Human Protein Atlas). Retinoic acid production by the HTPCs in vitro was confirmed (*n* = 2). Higher levels were found in the supernatant of the cells without FCS in the culture medium, i.e., a condition, in which also the levels of *ALDH1A1* were elevated. Table A3 (Appendix B) shows the comparison of our data (HTPC-1/HTPC-2; no FCS) with the data of Di Persio et al. [3] and Nie et al. [17].

### 3.6. Human Testicular Peritubular Cells Express Mesenchymal Stromal Cell Markers and Leydig Stem Cell Markers

**Figure 7:**
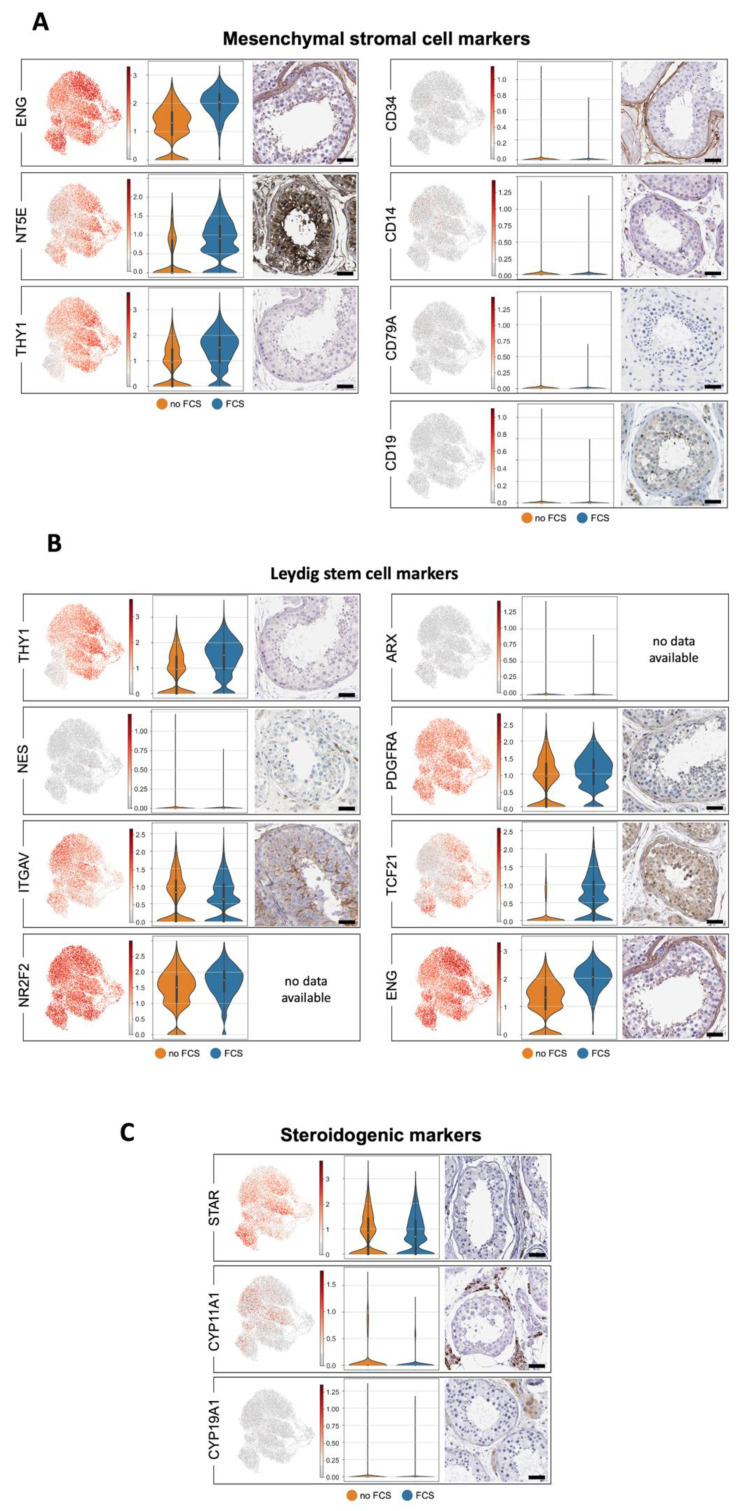
Markers of mesenchymal stromal cells, Leydig stem cells and steroidogenic enzymes. (**A**–**C**): UMAP plots of prototype smooth muscle markers in HTPCs (**left side**). Violin plots comparing expression with and without FCS of HTPC-1, (**middle**) and corresponding protein detection with HPA images (**right side**; scale bars = 50 µm). Please, note that abundances of some transcripts are affected by FCS. A comparison between HTPCs and ex vivo data is provided in Table A3 (Appendix B). (**A**) Transcripts of mesenchymal stromal cell markers with high expression levels of *ENG*, *NT5E* and *THY1*, while *CD34*, *CD14*, *CD79A* and *CD19* show weak or no expression. (**B**) Leydig stem cell markers are clearly expressed in HTPCs, except for *NES* and *ARX*. (**C**) The steroidogenic enzymes *STAR* and *CYP11A1* are expressed in HTPCs, while *CYP19A1* is not expressed in HTPCs.

Based on the minimal criteria for defining multipotent MSCs by Dominici et al. [45], MSCs must be plastic adherent when they are maintained in standard culture conditions, must express CD105 (ENG), CD73 (NT5E) and CD90 (THY1), and they must lack the expression of CD45 (PTPRC), CD34, CD14 or CD11B (ITGAM), CD79A or CD19 and HLA-DR (HLA-DRA/HLA-DRB1) surface molecules. These criteria are met by HTPCs, in general, although some cells were found to express *CD34*, *CD14*, *CD79A* or *CD19*. Of note, the expression of *ENG*, *NT5E* and *THY1* was increased when FCS was added to the culture medium (Figure 7). The available images of the immunostained peritubular cells that are provided in the Human Protein Atlas support this pattern of expression. Table A3 (Appendix B) shows the comparison of our data (HTPC-1/HTPC-2; no FCS) with the data of Di Persio et al. [3] and Nie et al. [17].

A third criterium, namely, that the MSCs must differentiate into osteoblasts, adipocytes and chondroblasts in vitro was not confirmed as our attempts to differentiate HTPCs to adipocytes and bone cells were not successful (Table A4, Appendix B).

The human peritubular wall is being considered to be a niche for Leydig stem cells (LSC). A number of markers for LSCs had been previously suggested in human and nonhuman species. They include THY1 (CD90), CD51 (ITGAV), COUP-TFII (NR2F2), PDGFRA, TCF21 and Endoglein (ENG) [46,47]. These were readily detected, yet Nestin (*NES*) and *ARX* were not readily found. The available images of the immunostained peritubular cells that are provided in the Human Protein Atlas support this expression (Figure 7).

Of note, *TCF21* expression was higher in the FCS-treated cultures, and the testicular cells with this marker have recently been identified to contribute to the somatic cells of the rodent testis, and they can regenerate adult Leydig cells in mice [34]. This led us to examine whether they may represent LSCs in humans, and we attempted to achieve differentiation [34]. It was not successful, and neither *INSL3* nor *LHR*, i.e., the prototype markers of the LSCs, became detectable (Table A4, Appendix B).

A group of HTPCs is characterized by the expression of enzymes involved in steroid production, i.e., *STAR* and *CYP11A1* (*SCC*). The corresponding proteins were not readily found in the images that were provided by the Human Protein Atlas collection, but the transcripts have been described by Di Persio et al. [3] and Nie et al. [17], indicating that the ability of HTPCs to produce sex steroids in situ must be considered (Figure 7). Table A3 (Appendix B) shows the comparison of our data (HTPC-1/HTPC-2; no FCS) with the data of Di Persio et al. [3] and Nie et al. [17].

## 4. Discussion

We compared the data from ex vivo-derived HTPCs, which recently became available [3,17] with our data, which were derived from isolated and cultured HTPCs. As the peritubular cells constitute only a small cell group within the human testis, the ex vivo studies are based on relatively few peritubular cells (several hundred), whereas we were able to study substantially more (more than 6000). We noted the strong overlap of the expressed genes between all of the data sets. While it is difficult to make generalized statements using only our small sample size, the strong overlap with the mentioned ex vivo studies, leads us to conclude that the isolated HTPCs represent a highly adequate cellular model, which allows for the study of the functions, regulation and plasticity of peritubular cells of the human testis, i.e., the least examined cells of the human testis. Hence, the results provide a thorough molecular signature of adult human testicular peritubular cells. We anticipate that the data that were obtained will be a resource for future studies.

While the overall difference between the ex vivo-derived HTPCs and the in vitro-derived cells were small, it cannot be ruled out that they could be due to isolation and cell culture. Yet, importantly, the direct comparison between the data from the two studies of the ex vivo-derived HTPCs [3,17] indicated differences which are in the same order of magnitude as the differences between our data and the ex vivo-studies. This therefore suggests that the observed differences may be due to technical issues, and more likely, to the cellular plasticity of the HTPCs, i.e., their general ability to perform phenotype switching.

Phenotype switching is an intrinsic and not-fully-understood ability of SMCs [37], which can present a number of cellular phenotypes, ranging from a contractile state to a secretory state and beyond. Such phenotypes, which can be adopted by and are reported for vascular SMCs, are macrophage-like, mesenchymal stem cell-like, myofibroblast-like and osteochondral-like phenotypes [37]. This topic is of particular interest in atherosclerosis research, and most of the data that are available stem from studies of vascular SMCs [48,49,50]. It is tempting to speculate that it may also be of relevance for the human testis, especially in male infertility, where the loss of the contractile markers of the peritubular cells and fibrosis of the tubular wall have been reported [14,15].

To explore the cellular plasticity of HTPCs, we exposed them to FCS. The addition of FCS caused cell proliferation, and as expected, the transcripts associated with cell cycle were altered. The treatment also revealed four distinct states of differentiation which were characterized by the expression of genes related to, e.g., “collagens”, “extracellular region”, “ECM”, “ER to Golgi transport” and others, thereby providing a proof of principle for cellular plasticity and functional heterogeneity of HTPCs.

Of note, while they are almost identical in their transcript expression, upon the commencement of the velocity analyses, the two samples (HTPC-1/HTPC-2) that have been examined here were not completely identical in this respect. The samples were from individual men, and it seems possible that differences are patient-related. The age differences between the donors (41 versus 48 years old) may also be involved. The fact that HTPCs and non-human primate testes-derived TPCs can age has been previously demonstrated, and insights into testicular and peritubular cell senescence have been gained [22,51]. It is therefore a possibility that the observed differences in the velocity analyses of HTPC-1 and HTPC-2 might indicate age-associated changes, specifically, in the ability of the HTPCs to undergo phenotypic switching. 

HTPCs were originally named SMCs, and later, they were also named myofibroblasts [52,53]. Our results shed a new light on their cellular identity. We compared the markers present in the HTPCs with the ones that have been described in other human SMC types [36]. Especially, the expression of *ACTA2*, *TAGLN* and *SMTN* provide support for the SMC character [36,54,55]. Testis specificity is indicated, among others, by the expression of steroid receptors and steroidogenic enzymes, e.g., *AR*, *STAR* and *CYP11A1*, which to our knowledge are not known in this combination in other human SMC types [56]. Of note, PDGFRB has been reported in some types of SMCs (i.e., vascular SMCs), but it is considered also to be a myofibroblast marker [55]. It has been identified in the HTPCs together with PDGFRA, and it was also detected in situ [3,17]. This receptor is down-regulated by the presence of FCS in the HTPCs, further indicating the dynamic and plastic nature of these cells. The changes associated with FCS in the medium indicated that the HTPCs are able to respond to stimulatory clues with an altered contractile phenotype and an altered receptor expression. Of note, other receptors, e.g., *AR*, *NR3C1* or *EGFR,* were not or not strongly affected by FCS.

PDGFRA and CD34, but of note not ACTA2, are thought to be typical for telocytes, and they have been reported in the human testis [57,58]. The present results, thus, do not indicate that they are present in the cultured HTPCs.

ECM is present between the layers of peritubular cells in the human testis, normally, and its composition and ECM amounts change in idiopathic male infertility when, for example, the DCN and BGN levels increase [10,40]. This most likely has consequences as, e.g., high testicular BGN levels can promote a pro-inflammatory response of HTPCs [40]. The ECM of the peritubular wall is produced by the HTPCs, and our data clearly support this notion and further reveal the repertoire of ECM components, which also includes collagens and other proteoglycans (these are not shown). The changes to *BGN* and *DCN* upon the addition of FCS indicate that HTPCs respond to clues with altered ECM production. 

The expression of the MSC markers (namely, *THY1* (*CD90*), *ENG* (*CD105*) and *NT5E* (*CD73*)) were also observed [45], prompting us to attempt differentiation studies. However, our attempts to differentiate the cultured HTPCs to bone or fat cells failed.

*TCF21* expression became evident. This factor, among others [46,47,59,60,61,62,63], was implicated with the LSCs, and this led us to attempt further differentiation studies. Following a recently published protocol [34], we performed such differentiation studies using HTPCs from two additional patients. However, this did not lead to the induction of a Leydig cell phenotype. The *STAR* levels were increased, but the markers *INSL3* and *LHR* remained undetectable. Hence, the potential of the HTPCs to differentiate into mature LCs remains to be further studied.

Of note, the HTPCs expressed *CYP11A1* and *STAR*. We have previously shown [41] that forskolin, when it is added to the HTPCs, increased *STAR* and *CYP11A1* levels and this resulted either in the induction or in the elevation of the secretion of pregnenolone, and in some cases also of progesterone. Hence, HTPCs have the intrinsic ability to produce these steroids, but they most likely cannot synthesize androgens.

It is possible that failure of HTPCs to differentiate into LCs or fat/bone cells could be due to the age of the donors and thus possible senescence-related changes of LSC and MSC [64,65]. This has not yet been reported with regard to LSCs, to our knowledge, yet there are reports indicating that the Leydig cell population decreases in the testes of old men [66]. As the HTPCs were from human donors, studies with HTPCs from young men are not readily possible and we, therefore, are not able to further investigate these points.

While HTPCs are known to be important for sperm transport [19], HTPCs also contribute to spermatogenesis. GDNF is a known growth factor, which is important for SSC renewal [43,44,67,68], and its expression was confirmed in our study. It appeared not to be affected by FCS. The results of our HTPC-study further led to the identification of unknown characteristics of HTPCs, namely, expression of *ALDH1A1* and *ALDH1A3*, i.e., enzymes that are involved in retinoic acid production. Of note, this expression was also detected in situ. The studies of two additional HTPC samples showed that retinoic acid is produced by these cells and that the levels are regulated by FCS. Whether retinoic acid that is derived from HTPCs may play a role in the regulation of human spermatogenesis [69,70] and/or in the regulation of the SMC phenotype of HTPCs [71], and/or in Sertoli cell GDNF synthesis [72] are possibilities that await further studies. That retinoic acid might target and be involved in the regulation of HTPCs is however indicated by the presence of respective receptors (*RXRA*/*RXRB*/*RARG*/*RARB*) in situ.

In conclusion, the results show that our model of cultured HTPCs is a unique and highly adequate cellular model for the study of the plasticity and function of human testicular peritubular cells, which allow one to gain insights into the otherwise inaccessible human male gonad. While the scRNAseq method-derived data do not allow us to deduce to the level of gene expression in individual cells, they provide a molecular signature of HTPCs. We anticipate that this signature will stimulate future studies and promote research to better understand human testicular function.

## Figures and Tables

**Table 1:** Smooth muscle cell marker transcripts in HTPCs and comparison with the data of ex vivo studies of Di Persio et al. [3] and Nie et al. [17]. √ indicates expression; ✕ indicates absence; √ when in brackets, indicates weak expression.

Gene Name	Description	HTPC-1	HTPC-2	TPCs of Nie et al. [17]	TPCs of Di Persio et al. [3]
*ACTA2*	Smooth-muscle actin	√	√	√	√
*MYH11*	Myosin heavy chain 11	√	√	√	√
*TAGLN*	Transgelin	√	√	√	√
*CNN1*	Calponin 1	√	√	✕	√
*CALD1*	Caldesmon 1	√	√	√	√
*SRF*	Serum response factor	√	√	√	√
*MYOCD*	Myocardin	(√)	✕	√	√
*VIM*	Vimentin	√	√	√	√
*DES*	Desmin	√	√	√	√
*SMTN*	Smoothelin	√	√	√	√
*PDGFRB*	Platelet derived growth factor receptor B	√	√	√	√

**Figure A2:**
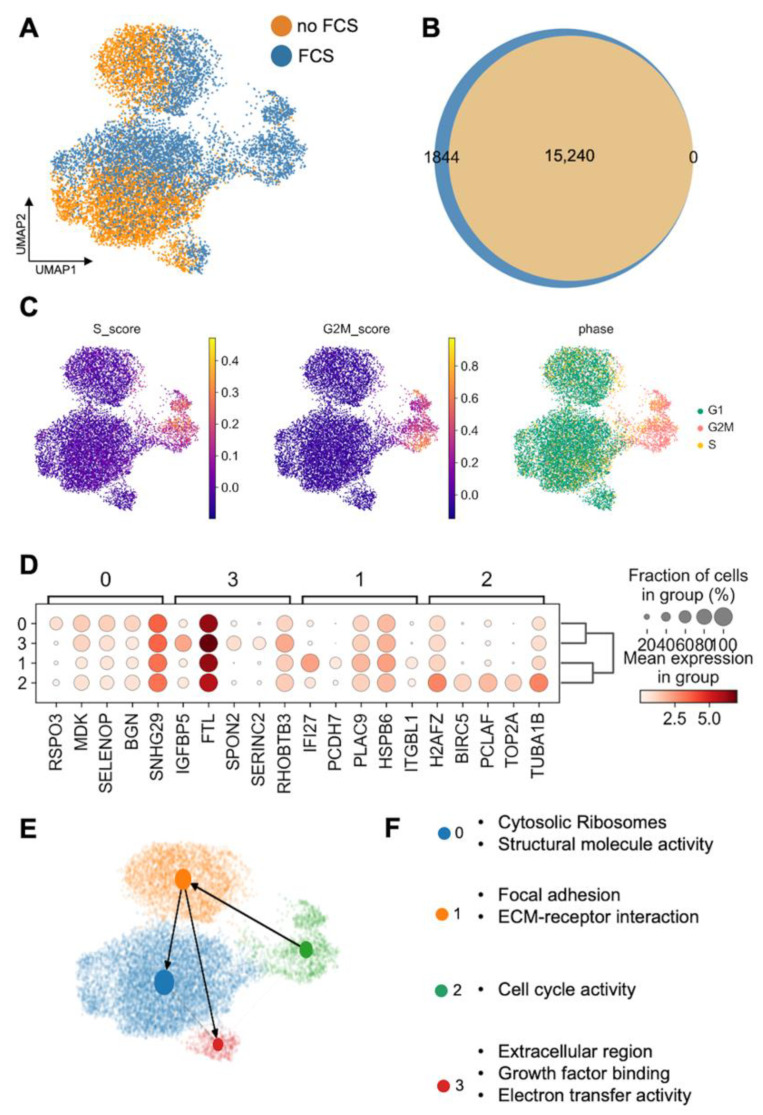
(**A**): UMAP plot of samples derived from HTPC-2 with and without FCS. (**B**): Venn diagram indicates overlap of both samples. The exposure of HTPCs to FCS results in the expression of 1844 additional genes. (**C**): Cell cycle stages [31] display one distinct proliferating cluster in the FCS sample. (**D–F**): Four distinct states of cellular differentiation were observed, indicating plasticity and proliferation. (**D**) Dot plot demonstrates expression of most abundant genes in each cluster. (**E**) Velocity analysis was embedded into the PAGA graph, which indicates trajectory of the proliferating cluster into cluster 1 and the two other functional subgroups. (**F**) Representative GO-terms after string analysis with upregulated genes.

## Data Availability

Raw and processed datasets of RNA single-cell sequencing studies have been deposited at NIH GEO (Gene Expression Omnibus): GSE212944, and they are available from of the date of publication.

## Appendix B

**Table A1:** Table of HPA-derived images, links and antibody information.

Gene Name	Link	Antibody
**Smooth muscle markers**		
*ACTA2*	https://images.proteinatlas.org/2/368_A_6_6.jpg (accessed on 15 October 2022)	Antibody CAB000002
*MYH11*	https://images.proteinatlas.org/15310/36933_A_6_6.jpg (accessed on 15 October 2022)	Antibody HPA015310
*TAGLN*	https://images.proteinatlas.org/1447/4295_A_4_6.jpg (accessed on 15 October 2022)	Antibody CAB001447
*CNN1*	https://images.proteinatlas.org/7/1341_A_6_6.jpg (accessed on 15 October 2022)	Antibody CAB000007
*CALD1*	https://images.proteinatlas.org/8066/21805_A_6_6.jpg (accessed on 15 October 2022)	Antibody HPA008066
*SRF*	https://images.proteinatlas.org/1819/7579_A_4_6.jpg (accessed on 15 October 2022)	Antibody HPA001819
*MYOCD*	https://images.proteinatlas.org/46485/102910_A_5_6.jpg (accessed on 15 October 2022)	Antibody CAB046485
*VIM*	https://images.proteinatlas.org/1762/7628_A_6_6.jpg (accessed on 15 October 2022)	Antibody HPA001762
*DES*	https://images.proteinatlas.org/34/2106_A_4_6.jpg (accessed on 15 October 2022)	Antibody CAB000034
*SMTN*	https://images.proteinatlas.org/51778/145653_A_6_6.jpg (accessed on 15 October 2022)	Antibody HPA051778
*PDGFRB*	https://images.proteinatlas.org/3842/10254_A_4_6.jpg (accessed on 15 October 2022)	Antibody CAB003842
**Producers of ECM**		
*DCN*	https://images.proteinatlas.org/17118/38996_A_6_6.jpg (accessed on 15 October 2022)	Antibody CAB017118
*BGN*	https://images.proteinatlas.org/3157/9472_A_4_6.jpg (accessed on 15 October 2022)	Antibody HPA003157
*COL1A1*	https://images.proteinatlas.org/11795/32976_A_6_6.jpg (accessed on 15 October 2022)	Antibody HPA011795
**Receptors**		
*AR*	https://images.proteinatlas.org/1/135_A_6_6.jpg (accessed on 15 October 2022)	Antibody CAB000001
*NR3C1*	https://images.proteinatlas.org/4248/13599_A_6_6.jpg (accessed on 15 October 2022)	Antibody HPA004248
*ESR1*	https://images.proteinatlas.org/55099/122099_A_4_6.jpg (accessed on 15 October 2022)	Antibody CAB055099
*PGR*	https://images.proteinatlas.org/4751/15549_A_6_6.jpg (accessed on 15 October 2022)	Antibody HPA004751
*GPER1*	https://images.proteinatlas.org/27052/56949_A_6_6.jpg (accessed on 15 October 2022)	Antibody HPA027052
**Growth factor receptors**		
*EGFR*	https://images.proteinatlas.org/35/895_A_6_6.jpg (accessed on 15 October 2022)	Antibody CAB000035
*PDGFRA*	https://images.proteinatlas.org/18143/43112_A_5_6.jpg (accessed on 15 October 2022)	Antibody CAB018143
**Ion channels**		
*TRPV2*	https://images.proteinatlas.org/44993/102399_A_5_6.jpg (accessed on 15 October 2022)	Antibody HPA044993
*P2RX4*	https://images.proteinatlas.org/39494/85835_A_5_6.jpg (accessed on 15 October 2022)	Antibody HPA039494
*P2RX7*	https://images.proteinatlas.org/42013/103582_A_5_6.jpg (accessed on 15 October 2022)	Antibody HPA042013
**Aldehyde dehydrogenases**		
*ALDH1A1*	https://images.proteinatlas.org/2123/8966_A_6_6.jpg (accessed on 15 October 2022)	Antibody HPA002123
*ALDH1A2*	https://images.proteinatlas.org/10022/24728_A_6_6.jpg (accessed on 15 October 2022)	Antibody HPA010022
*ALDH1A3*	https://images.proteinatlas.org/46271/105391_A_6_6.jpg (accessed on 15 October 2022)	Antibody HPA046271
**Mesenchymal stromal cell markers**		
*ENG/CD105*	https://images.proteinatlas.org/67440/156475_A_5_6.jpg (accessed on 15 October 2022)	Antibody HPA067440
*CD73/NT5E*	https://images.proteinatlas.org/17357/41086_A_5_6.jpg (accessed on 15 October 2022)	Antibody HPA017357
*CD90/THY1*	https://images.proteinatlas.org/68243/152840_A_6_6.jpg (accessed on 15 October 2022)	Antibody CAB068243
*CD34*	https://images.proteinatlas.org/36722/122320_A_5_6.jpg (accessed on 15 October 2022)	Antibody HPA036722
*CD14*	https://images.proteinatlas.org/72865/157660_A_6_6.jpg (accessed on 15 October 2022)	Antibody CAB072865
*CD79A*	https://images.proteinatlas.org/19/247_A_6_6.jpg (accessed on 15 October 2022)	Antibody CAB000019
*CD19*	https://images.proteinatlas.org/16110/36120_A_6_6.jpg (accessed on 15 October 2022)	Antibody CAB016110
**Leydig cells factors**		
*THY1*	https://images.proteinatlas.org/68243/152840_A_6_6.jpg (accessed on 15 October 2022)	Antibody CAB068243
*NES*	https://images.proteinatlas.org/7007/20723_A_4_6.jpg (accessed on 15 October 2022)	Antibody HPA007007
*CD51/ITGAV*	https://images.proteinatlas.org/2499/6125_A_4_6.jpg (accessed on 15 October 2022)	Antibody CAB002499
*PDGFRA*	https://images.proteinatlas.org/18143/43112_A_5_6.jpg (accessed on 15 October 2022)	Antibody CAB018143
*TCF21*	https://images.proteinatlas.org/13189/60539_A_5_6.jpg (accessed on 15 October 2022)	Antibody HPA013189
*ENG/CD105*	https://images.proteinatlas.org/67440/156475_A_5_6.jpg (accessed on 15 October 2022)	Antibody HPA067440
**Steroidogenic markers**		
*STAR*	https://images.proteinatlas.org/27318/166039_A_4_6.jpg (accessed on 15 October 2022)	Antibody HPA027318
*CYP11A1*	https://images.proteinatlas.org/16436/142441_A_4_6.jpg (accessed on 15 October 2022)	Antibody HPA016436
*CYP19A1*	https://images.proteinatlas.org/355/2047_A_5_6.jpg (accessed on 15 October 2022)	Antibody CAB000355
**Retinoic acid & Retinoid X receptors**		
*RARB*	https://images.proteinatlas.org/4174/169826_A_5_6.jpg (accessed on 15 October 2022)	Antibody HPA004174
*RARG*	https://images.proteinatlas.org/53883/121805_A_5_6.jpg (accessed on 15 October 2022)	Antibody HPA053883
*RXRA*	https://images.proteinatlas.org/4565/13151_A_5_6.jpg (accessed on 15 October 2022)	Antibody CAB004565
*RXRB*	https://images.proteinatlas.org/2003/5372_A_6_6.jpg (accessed on 15 October 2022)	Antibody CAB002003

**Table A2:** List of oligonucleotide primer pairs used for qPCR amplification.

Gene Name	Nucleotide Sequence	Amplicon Size	Annealing Temperature
*ALPL*	5′-GGA TGG GTG TCT CCA CAG TG-3′ 5′-GCT CTT CCA GGT GTC AAC GA-3′	593 bp	60 °C
*COMP*	5′-GGA TGC CTG TGA CAA CTG TC-3′ 5′-AAG GCC CTG AAG TCG GTG AG-3′	390 bp	62 °C
*INSL3*	5′-GGG AGT CTC ACT CTG TTT CCC-3′ 5′-CAC TGT AGC AAC TCA CAT CGC-3′	108 bp	60 °C
*LHR*	5′-TGG AAA TGG ATT TGA AGA AGT ACA-3′ 5′-CAC GGA AGG CTC CAT TGT-3′	113 bp	60 °C
*PLIN1*	5′-ACC TGC GAA TGC TTC CAG AA-3′ 5′-CAG GGG CTG ACT CTT CCT TG-3′	508 bp	60 °C
*STAR*	5′-ACG TGG ATT AAC CAG GTT CG-3′ 5′-CAG CCC TCT TGG TTG CTA AG-3′	149 bp	58 °C

**Table A3:** Transcript comparison of our data (HTPC-1/HTPC-2) with the data of Di Persio et al. [3] and Nie et al. [17]. √ indicates expression, ✕ indicates absence, √ when in brackets, indicates weak expression.

Gene Name	Description	HTPC-1	HTPC-2	TPCs of Nie et al. [17]	TPCs of Di Persio et al. [3]
*ACTA2*	Smooth-Muscle Actin	√	√	√	√
*ALDH1A1*	Aldehyde Dehydrogenase 1 Family Member A1	√	√	√	√
*ALDH1A2*	Aldehyde Dehydrogenase 1 Family Member A2	(√)	✕	√	(√)
*ALDH1A3*	Aldehyde Dehydrogenase 1 Family Member A3	√	√	√	√
*AR*	Androgen Receptor	√	√	√	√
*ARX*	Aristaless Related Homeobox	✕	(√)	√	√
*BGN*	Biglycan	√	√	√	√
*CALD1*	Caldesmon 1	√	√	√	√
*CD14*	Monocyte Differentiation Antigen CD14	(√)	✕	√	✕
*CD19*	B-Lymphocyte Surface Antigen B4	✕	✕	✕	✕
*CD34*	Hematopoietic Progenitor Cell Antigen CD34	✕	✕	√	√
*CD79A*	B-Cell Antigen Receptor Complex-Associated Protein Alpha Chain	✕	✕	✕	✕
*CNN1*	Calponin 1	√	√	✕	√
*COL1A1*	Collagen Type I Alpha 1 Chain	√	√	√	√
*CXCL12*	C-X-C Motif Chemokine Ligand 12	√	√	√	√
*CYP11A1*	Cholesterol Side-Chain Cleavage Enzyme	√	√	√	√
*CYP19A1*	Aromatase	✕	✕	✕	✕
*DCN*	Decorin	√	√	√	√
*DES*	Desmin	√	√	√	√
*EGFR*	Epidermal Growth Factor Receptor	√	√	√	√
*ENG*	Endoglin	√	√	√	√
*ESR1*	Estrogen Receptor	√	√	√	(√)
*GDNF*	Glial Cell Derived Neurotrophic Factor	√	(√)	✕	✕
*GPER*	G-Protein coupled Estrogen Receptor	√	√	✕	✕
*ITGAV*	Integrin Subunit Alpha V	√	√	√	√
*LIF*	Leukemia Inhibitory Factor	(√)	(√)	✕	✕
*MYH11*	Myosin Heavy Chain 11	√	√	√	√
*MYOCD*	Myocardin	(√)	✕	√	√
*NES*	NES	✕	✕	√	(√)
*NGF*	Nerve Growth Factor	√	√	✕	✕
*NR2F2*	NR2F2	√	√	√	√
*NR3C1*	Glucocorticoid Receptor	√	√	√	√
*NT5E*	Ecto-5’-Nucleotidase	√	√	√	√
*P2RX4*	Purinergic Receptor P2X4	√	√	√	√
*P2RX7*	Purinergic Receptor P2X7	√	√	√	√
*PDGFRA*	Platelet Derived Growth Factor Receptor Alpha	√	√	√	√
*PDGFRB*	Platelet Derived Growth Factor Receptor B	√	√	√	√
*PGR*	Progesterone Receptor	√	√	√	√
*SMTN*	Smoothelin	√	√	√	√
*SRF*	Serum Response Factor	√	√	√	√
*STAR*	Steroidogenic Acute Regulatory Protein	√	√	√	√
*TAGLN*	Transgelin	√	√	√	√
*TCF21*	Transcription Factor 21	√	√	√	√
*THY1*	Thy-1 Cell Surface Antigen	√	√	√	√
*TRPV2*	Transient Receptor Potential Cation Channel Subfamily V Member 2	√	√	√	√
*VIM*	Vimentin	√	√	√	√

**Table A4:** Summary of differentiation studies. Additional HTPCs (from 2 to 4 donors) were treated as described in Materials and Methods and analyzed for Leydig cell-, osteoblast- and adipocyte-markers (Ctrl. = control group; Diff. = respective differentiation medium; n.d. = transcript not detectable; +, ++, +++ = indicates low, medium and high expression levels; Alc. ph. = alkaline phosphatase).

Leydig Cell Differentiation						
Donor	qPCR analysis					
	*LHR*		*INSL3*		*STAR*	
1	Ctrl.	Diff.	Ctrl.	Diff.	Ctrl.	Diff.
2	n.d.	n.d.	n.d.	n.d.	+	++
3	n.d.	n.d.	n.d.	n.d.	+	++
4	n.d.	n.d.	n.d.	n.d.	+	++
Osteoblast Differentiation						
Donor	qPCR analysis				Alc. ph. staining	
	*COMP*		*ALPL*			
	Ctrl.	Diff.	Ctrl.	Diff.	Ctrl.	Diff.
1	+	+++	+	+	n.d.	n.d.
2	+	+++	+	+	n.d.	n.d.
Adipocyte Differentiation						
Donor	qPCR analysis				Oil red staining	
	*PLIN1*					
	Ctrl.		Diff.		Ctrl.	Diff.
1	n.d.		n.d.		n.d.	n.d.
2	+		+		n.d.	n.d.
3	+		+			
4	+		+			
